# Improved xylose and arabinose utilization by an industrial recombinant *Saccharomyces cerevisiae *strain using evolutionary engineering

**DOI:** 10.1186/1754-6834-3-13

**Published:** 2010-06-15

**Authors:** Rosa Garcia Sanchez, Kaisa Karhumaa, César Fonseca, Violeta Sànchez Nogué, João RM Almeida, Christer U Larsson, Oskar Bengtsson, Maurizio Bettiga, Bärbel Hahn-Hägerdal, Marie F Gorwa-Grauslund

**Affiliations:** 1 Department of Applied Microbiology, Lund University, PO Box 124, SE-22100 Lund, Sweden; 2 Center for Microbial Biotechnology, Department of Systems Biology, Technical University of Denmark, Soltofts plads, 2800 Kgs Lyngby, Denmark; 3 Centro de Recursos Microbiológicos (CREM), Departamento de Ciências da Vida, Faculdade de Ciências e Tecnologia, Universidade Nova de Lisboa, 2829-516 Caparica, Portugal; 4 Laboratório Nacional de Energia e Geologia, I.P., Unidade de Bioenergia, Estrada do Paço do Lumiar 22, 1649-038, Lisboa, Portugal; 5 Carlsberg Research Center, Gamle Carlsberg vej 10, DK-2500 Valby, Denmark; 6 Department of Chemistry, Biotechnology and Food Science Norwegian University of Life Sciences, PO Box 5003, N-1432 Ås, Norway

## Abstract

**Background:**

Cost-effective fermentation of lignocellulosic hydrolysate to ethanol by *Saccharomyces cerevisiae *requires efficient mixed sugar utilization. Notably, the rate and yield of xylose and arabinose co-fermentation to ethanol must be enhanced.

**Results:**

Evolutionary engineering was used to improve the simultaneous conversion of xylose and arabinose to ethanol in a recombinant industrial *Saccharomyces cerevisiae *strain carrying the heterologous genes for xylose and arabinose utilization pathways integrated in the genome. The evolved strain TMB3130 displayed an increased consumption rate of xylose and arabinose under aerobic and anaerobic conditions. Improved anaerobic ethanol production was achieved at the expense of xylitol and glycerol but arabinose was almost stoichiometrically converted to arabitol. Further characterization of the strain indicated that the selection pressure during prolonged continuous culture in xylose and arabinose medium resulted in the improved transport of xylose and arabinose as well as increased levels of the enzymes from the introduced fungal xylose pathway. No mutation was found in any of the genes from the pentose converting pathways.

**Conclusion:**

To the best of our knowledge, this is the first report that characterizes the molecular mechanisms for improved mixed-pentose utilization obtained by evolutionary engineering of a recombinant *S. cerevisiae *strain. Increased transport of pentoses and increased activities of xylose converting enzymes contributed to the improved phenotype.

## Background

Lignocellulosic feedstocks, in particular agricultural wastes and hardwoods, contain a large fraction of the pentose sugars D-xylose and L-arabinose [1]. Therefore, cost-efficient fermentation of lignocellulosic hydrolysates to ethanol requires a micro-organism that can ferment pentose sugars to ethanol with high yield and productivity. *Saccharomyces cerevisiae *is the conventional microorganism for ethanol production, but it cannot naturally ferment pentose sugars. Consequently, various metabolic engineering strategies have been employed to express both fungal and bacterial genes encoding either D-xylose or L-arabinose utilizing pathways in *S. cerevisiae *(reviewed recently in, for example, [2,3]). Despite these efforts, the sole heterologous expression of these genes was not sufficient to enable efficient fermentation to ethanol and additional modifications were required to overcome limitations in the conversion of pentoses.

**Figure 1:**
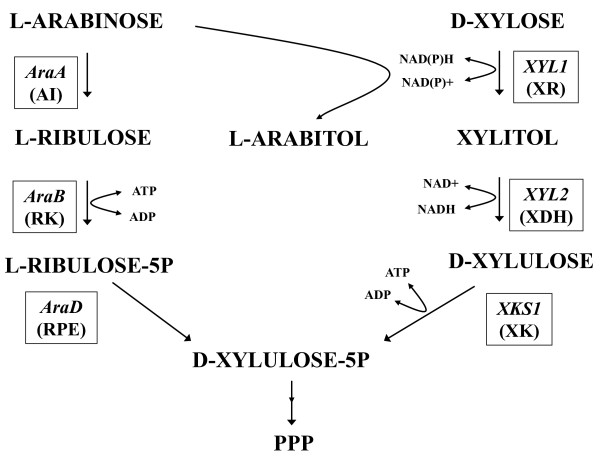
**D-xylose and L-arabinose utilization pathways engineered in the *Saccharomyces cerevisiae *strains TMB3061, TMB3063 and TMB3130**. Fungal D-xylose utilizing pathway: *Pichia stipitis *D-xylose reductase (XR), *XYL1*; *P. stipitis *D-xylitol dehydrogenase (XDH), *XYL2*; *S. cerevisiae *D-xylulose kinase (XK); *XKS1*. Bacterial L-arabinose utilizing pathway: *Bacillus subtilis *L-arabinose isomerase (AI), *AraA*; *Escherichia coli *L-ribulokinase (RK), *AraB*; *E. coli *L-ribulose-5-P 4 epimerase (RPE), *AraD*.

Using isogenic *S. cerevisiae *strain background, higher specific ethanol productivity from xylose was achieved in the strain expressing the xylose reductase (XR) and xylitol dehydrogenase (XDH) pathway (Figure 1), whereas higher ethanol yield was obtained when expressing the fungal xylose isomerase pathway [4]. With arabinose, the first functional bacterial arabinose pathway (Figure 1) expressed in *S. cerevisiae *combined the *Bacillus subtilis AraA *and *Escherichia coli AraB *and *AraD *[5]. Later studies have used homologous genes from *Lactobacillus plantarum *[6] or *B. licheniformis AraA *[7] to improve arabinose utilization. The fungal arabinose-utilization pathway has been also engineered in *S. cerevisiae *strains [8] enabling growth on arabinose.

Ethanol productivity would benefit from the design of stable *S. cerevisiae *strains that would co-consume xylose and arabinose, since both sugars are present in lignocellulosic feedstock. Co-expression of arabinose and xylose pathways in *S. cerevisiae *has been reported [6,9,10]. However, so far there is only one example of chromosomal integration of xylose and arabinose pathway genes in *S. cerevisiae *[10]. In these engineered industrial strains (named TMB3061 and TMB3063) that carry the *Pichia stipitis *xylose pathway combined with the *B. subtilis - E. coli *arabinose pathway (Figure 1), the consumption of arabinose and xylose was low and ethanolic fermentation was limited by extensive arabitol formation from XR enzyme [10] (Figure 1).

Evolutionary engineering (reviewed by [11]) complements rationally designed genetic engineering approaches. In the field of pentose fermentation, several examples of evolved *S. cerevisiae *strains that are improved for either xylose [12-15] or arabinose [5,6] fermentation have been reported. One more recent study describes the evolution of a *S. cerevisiae *strain towards both xylose and arabinose utilization by an haploid laboratory strain harbouring xylose and arabinose genes on multicopy plasmids [16].

In the present contribution, we report on an evolutionary engineering study to improve mixed sugar fermentation by the industrial polyploid *S. cerevisiae *strain TMB3061 previously engineered with integrated genes for xylose and arabinose co-utilization [10]. Carbon-limited continuous culture with xylose and arabinose and increasing dilution rate was used to select for a population with improved growth on a mixture of xylose and arabinose sugars. One isolate, TMB3130, was further physiologically characterized.

## Results

### Evolutionary engineering for improved xylose and arabinose co-utilization

The industrial *S. cerevisiae *strain TMB3061 (Table 1) that overexpresses *B. subtilis AraA*, *E. coli AraB *and *AraD*, *P. stipitis XYL1 *and *XYL2 *and *S. cerevisiae XKS1 *[10] (Figure 1) was used to initiate the evolution in continuous culture under aerobic conditions using xylose and arabinose as limiting carbon sources. The feed initially contained 10 g L^-1 ^xylose as the sole carbon source and it was changed to a mixture of 5 g L^-1 ^xylose and 5 g L^-1 ^arabinose after more than 400 h, equivalent to 20 generations. The dilution rate was manually increased whenever a steady-state (assessed by stable cell concentration) was reached or decreased when washout was observed.

**Table 1:** Description and aerobic growth on pentoses for the *Saccharomyces cerevisiae *strains used in the study

Strains	Relevant genotype/phenotype	Abbreviation	Maximum specific growth rate (h^-1^)		Maximum OD_620nm_ (average)		Strain reference
				
			Xylose	Arabinose	Xylose	Arabinose	
**TMB3400**	*HIS3::ADH1p- XYL1-ADH1t, PGKp-XYL2-PGKt, pPGK-XKS1-tPGK*	X	0.129 ± 0.010	0.002 ± 0.000	19.1	0.5	[31]

**TMB3061**	**TMB3400**, *TRP1:: YlpAraB KanMX*, *NTS2::HXT7p-AraA-CYC1t *and *NTS2:: HXT7p-AraD-CYC1t, KanMX*	**X+A**	0.016 ± 0.000	0.014 ± 0.000	11.1	5.1	[10]

**TMB3063**	**TMB3061**, additional copies of *NTS2*::*HXT7*p-*AraA*-*CYC1*t	**X+A+A**	0.027 ± 0.000	0.033 ± 0.000	12.1	5.0	[10]

**TMB3130**	**TMB3061**, evolved on xylose and arabinose	**X+A evolved**	0.054 ± 0.003	0.027 ± 0.010	23.3	21.5	This study

Growth parameters were determined under aerobic conditions on yeast nitrogen base (YNB) medium with 50 g L^-1 ^arabinose or 50 g L^-1 ^xylose. Cell pre-cultures for the inoculum were grown on YNB medium with 20 g L^-1 ^glucose.

OD, optical density.

After approximately 65 generations, the dilution rate had been increased from 0.02 to 0.06 h^-1 ^and the cell mass in the continuous culture reached an optical density (OD _620 nm_) higher than 20 as compared to initial values of 2-8 at the beginning of the experiment. A sample of the cell population after 70 generations was then chosen for further analysis.

### Isolation of the evolved strain TMB3130

The population harvested from the continuous culture was sequentially grown in shake flask cultures containing first arabinose and then xylose in order to select for isolates that grew efficiently on both carbon sources. Cells from the shake flask culture on xylose were streaked on yeast peptone glucose (YPD) for isolation of single colonies. Each colony was re-streaked on yeast nitrogen base (YNB) plates containing xylose or arabinose as sole carbon source and 20 selected isolates able to grow efficiently on both pentoses were selected. Further analysis of 11 isolates was conducted in YNB liquid cultures where the specific growth rate (μ_max_) and the final biomass concentration (maximum OD _620 nm_) were compared to identify the best-growing isolates.

At least a twofold increase in maximum OD_620 nm _was obtained for all isolates as compared to the parental strain TMB3061 when grown on xylose (data not shown)_. _On the contrary, the ability to grow on arabinose appeared to be very divergent between the isolates, ranging from μ_max _0.020 to 0.041. While most isolates reached the stationary phase at an OD_620 nm _between 1.2 and 2.2 in medium with arabinose, two isolates continued growing until an OD_620 nm _of about 16. When these two isolates had been transferred several times to fresh arabinose medium, μ_max _reached 0.040 h^-1 ^for both isolates indicating that μ_max _on arabinose had been increased threefold compared with the parental strain TMB3061 (μ_max _0.014 h^-1^) (Table 1).

One of the two isolates was chosen for further characterization and named TMB3130 (Table 1). Analytical polymerase chain reaction (PCR) of the overexpressed *XKS1 *confirmed that TMB3130 originated from the recombinant strain TMB3061.

### Aerobic growth on pentose sugars

The parental strain, TMB3061, and the newly isolated TMB3130 were compared with respect to aerobic growth on xylose and arabinose. The comparison also included: (i) TMB3400 which only harbours the heterologous xylose utilizing pathway and is the parental strain of TMB3061; and (ii) TMB3063 that is based on TMB3061 but has additional copies of *AraA *(Table 1) [10]. All strains were pre-grown on glucose before determination of μ_max _on pentose sugars.

**Figure 2:**
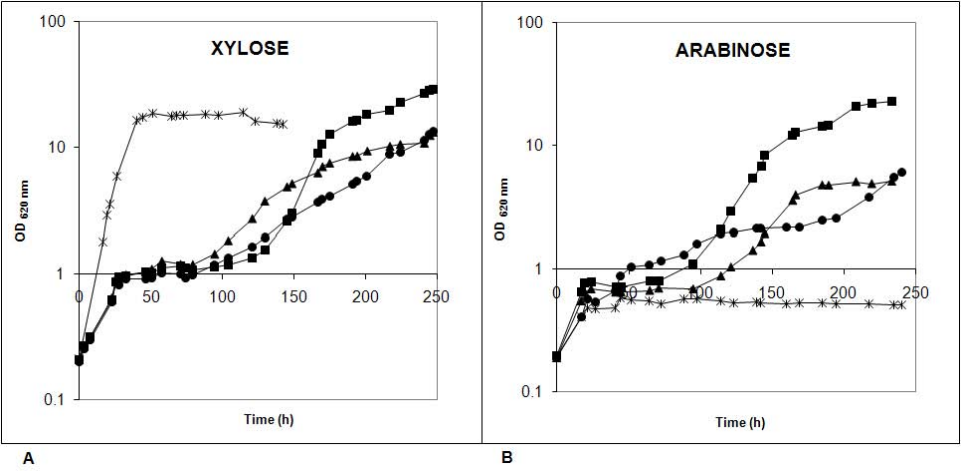
**Aerobic growth of *Saccharomyces cerevisiae *strains on pentose sugars**. (A) Yeast nitrogen base (YNB) medium with 50 g L^-1 ^xylose and (B) YNB medium with 50 g L^-1 ^arabinose. Cells were pre-grown on YNB medium with 20 g L^-1 ^glucose.  TMB3400 (X) (asterisk);  TMB3061 (X+A) (filled circle); TMB3063 (X+A+A) (filled triangle); and TMB3130 (X+A evolved) (filled square).

The evolved strain TMB3130 displayed an enhanced aerobic growth rate on xylose (three- to fourfold) and on arabinose (twofold) compared with its parental strain TMB3061 (Table 1). However, the μ_max _on xylose was still twofold lower than that of the xylose-utilizing TMB3400, the xylose-utilizing parental strain of TMB3061 [10] (Table 1). The lag-phase on pentoses of the two xylose and arabinose co-consuming strains was also longer than that of TMB3400 (Figure 2) and, thus, appeared to be related to the introduction of the arabinose-utilizing pathway. However, after adaptation, the novel isolate TMB3130 reached the highest final biomass concentration in both xylose containing-medium and in arabinose-containing medium (Table 1; Figure 2).

### Anaerobic co-fermentation of glucose, xylose and arabinose

Mixed sugar fermentation with the evolved isolate TMB3130 was compared with the xylose-consuming strain TMB3400 and with the xylose- and arabinose co-consuming strain TMB3063 (Table 2). Glucose was included in the medium together with xylose and arabinose to allow anaerobic growth at the start of the fermentation.

**Table 2:** Anaerobic co-fermentation on 20 g L^-1 ^glucose, 20 g L^-1 ^xylose and 20 g L^-1 ^arabinose on defined medium

Strains	*q *(consumption rate) in g (g cell h)^-1^		g sugar left at 120 h		*Y *arabitol (g arabitol. g consumed arabinose^-1^)	*Y *(g. g consumed xylose^-1^)			
			
	Xylose	Arabinose	Xylose	Arabinose		Y xylitol	Y glycerol	Y acetate	Y ethanol
**TMB3400**	0.07 ± 0.00	0.01 ± 0.01	0.23 ± 0.06	18.40 ± 0.12	0.94 ± 0.02	0.42 ± 0.01	0.09 ± 0.01	0.02 ± 0.00	0.22 ± 0.01

**TMB3063**	0.06 ± 0.02	0.05 ± 0.02	9.71 ± 1.35	13.97 ± 1.59	1.03 ± 0.08	0.21 ± 0.01	0.03 ± 0.00	0.10 ± 0.09	0.24 ± 0.03

**TMB3130**	0.09 ± 0.02	0.10 ± 0.04	2.11 ± 0.22	1.61 ± 2.28	0.95 ± 0.05	0.31 ± 0.01	0.04 ± 0.00	0.08 ± 0.02	0.29 ± 0.12

The consumption rates of xylose and arabinose as well as the yields of arabitol, xylitol, glycerol, ethanol and acetate were calculated from the pentose phase after glucose depletion. The cell dry weight at the end of fermentation was used for the calculation of the specific consumption rates. TMB3400 only harbours the heterologous xylose-utilizing pathway; TMB3063 is a derivative from TMB3061 (that contains the xylose and arabinose pathways) with extra-copies of *AraA *gene; TMB3130 is evolved from TMB3061.

**Figure 3:**
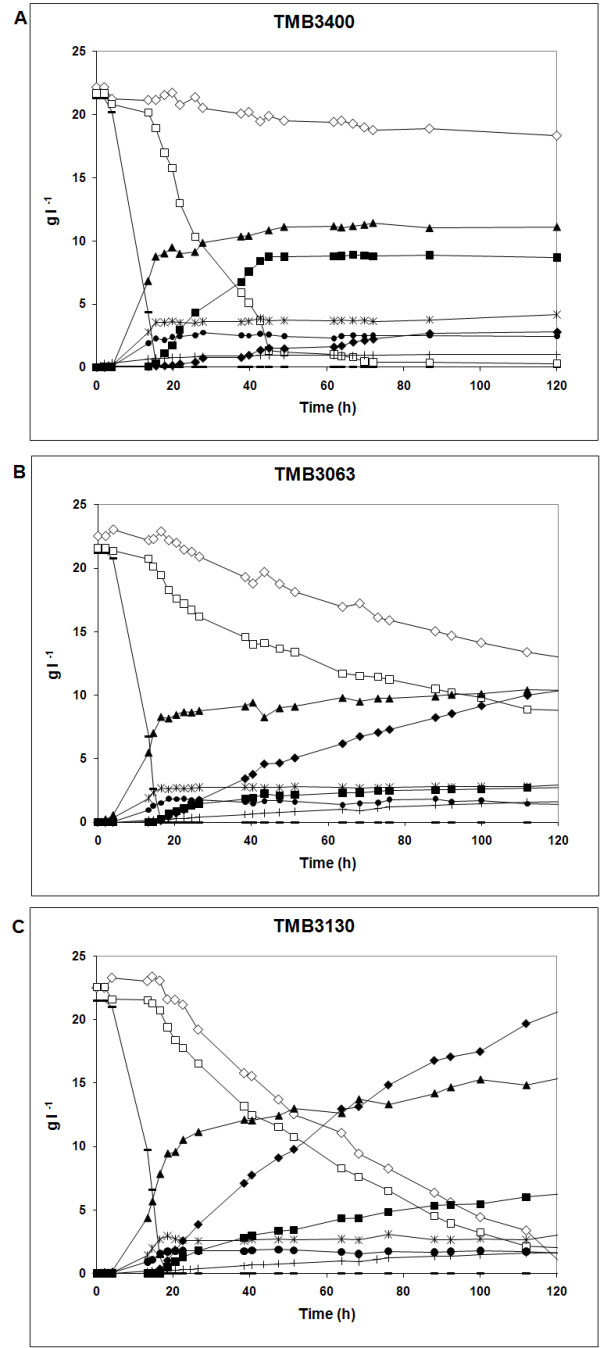
**Anaerobic co-fermentation on defined medium with 20 g L^-1 ^glucose, 20 g L^-1 ^xylose and 20 g L^-1 ^arabinose**. Cells for the inocula were pre-grown on defined medium with 20 g L^-1 ^glucose. The strains used were *Saccharomyces cerevisiae ***(A) **TMB3400, **(B) **TMB3063 and **(C) **TMB3130. Symbols:  glucose (minus symbol), xylose (open square), arabinose (open diamond), xylitol (filled square), arabitol (filled diamond), ethanol (filled triangle), biomass (filled circle), glycerol (asterisk), acetate (plus symbol).

Xylose and arabinose consumption rates were calculated during the pentose phase after exhaustion of glucose. The specific consumption rates of xylose and arabinose (Table 2) were the highest for the evolved strain TMB3130 and similar for the two pentose sugars (0.9-0.10 g (g cell h)^-1^). For the two other strains, the xylose consumption rate was higher than the arabinose consumption rate. Almost all xylose and arabinose were consumed by the evolved strain TMB3130, while 46% of xylose and 67% of arabinose were left in the medium by TMB3063. TMB3400 consumed almost all xylose but no arabinose, in accordance with its genetic make-up (Tables 1 and 2; Figure 3).

At the metabolite level, the ethanol yield was the highest in the evolved strain (0.29 g ethanol. g consumed xylose^-1^; Table 2). Almost all arabinose was converted to arabitol in the three strains (Table 2). TMB3400 (X) had the highest xylitol yield 0.42 (g xylitol. g xylose consumed)^-1 ^while TMB3063 (X+A+A) and TMB3130 (X+A evolved) had xylitol yields of 0.21 and 0.31, respectively. The glycerol yield from consumed xylose was higher for TMB3400 (0.09 g glycerol. g consumed xylose^-1^) and the lowest for TMB 3063 and TMB3130 (0.03 and 0.04 g glycerol. g consumed xylose^-1^, respectively; Table 2). In contrast, the acetate yield from consumed xylose was lower for TMB3400 (0.02 g acetate. g consumed xylose^-1^) and higher for TMB3063 (0.10 g acetate. g consumed xylose^-1^) and TMB3130 (0.08 g acetate. g consumed xylose^-1^; Table 2).

### Enzymatic activities of the xylose and arabinose pathways

XR and XDH activities of glucose-grown strain TMB3130 (X+A evolved) were compared to those previously obtained with glucose grown cells for the parental strain TMB3061 (X+A) and strains TMB3400 (X) and TMB3063 (X+A+A) [10]. XR and XDH activities had significantly increased in the evolved isolate TMB3130, five- to eightfold for XR and two- to 3.5-fold for XDH as compared to the activities measured in the other three strains (Table 3).

**Table 3:** Xylose reductase (XR) and xylitol dehydrogenase (XDH) activity (U.mg protein^- 1^) of crude protein extracts from cells grown in defined medium with 20 g L^-1 ^glucose

Strain	XR activity **U mg**^-1 ^**protein**	XDH activity **U mg**^-1 ^**protein**
**TMB 3400**	0.09 ± 0.00 (*)	0.59 ± 0.08 (*)

**TMB3061**	0.13 ± 0.01 (*)	0.52 ± 0.03 (*)

**TMB 3063**	0.10 ± 0.01 (*)	0.81 ± 0.08 (*)

**TMB 3130**	0.72 ± 0.06	1.85 ± 0.61

*Taken from [10]

TMB3400 only harbours the heterologous xylose-utilizing pathway; TMB3063 is a derivative from TMB3061 (that contains the xylose and arabinose pathways) with extra-copies of *AraA *gene; TMB3130 is evolved from TMB3061.

It was not possible to measure enzymatic activities of the arabinose pathways due to lack of commercially available compounds for the assays.

### Sequencing of genes from the xylose and arabinose pathways

The genes encoding the heterologous arabinose and xylose pathways in the strains TMB3061 and TMB3130 were sequenced and no difference in nucleotide sequence was detected between the two strains. However, the *B. subtilis AraA *sequence of both TMB3130 and TMB3061 was found to contain nine point mutations compared with the original *B. subtilis *sequence found in GeneBank. Three of these mutations were non-silent, common for both strains and were positioned in aminoacids 201 (Phe → Leu), 366 (Ser → Pro) and 378 (Leu → Phe). Also the *E. coli AraB *sequence of TMB3061 and TMB3130 had three point mutations compared with the original *E. coli *sequence of which one was non-silent in position 121 (Asp → Asn) [5]. In both strains the *P. stipitis XYL2 *gene contained four silent point mutations.

### Assessment of transport kinetics

**Figure 4:**
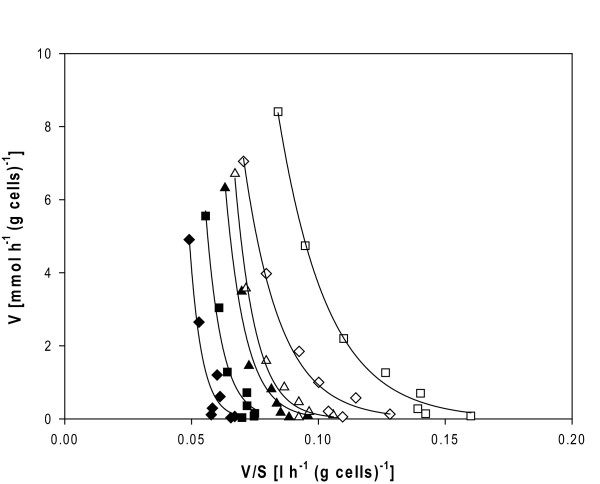
**D-[U-^14^C] xylose initial uptake rates in *Saccharomyces cerevisiae* strains engineered for xylose and arabinose utilization**. Eadie-Hofstee plot of D-[U-^14^C]xylose initial uptake rates in *S. cerevisiae *cells (TMB3400 (X), triangles; TMB3061 (X+A), diamonds; TMB3130 (X+A evolved), squares) grown in D-glucose (filled symbols) or D-xylose (open symbols).

*S. cerevisiae *strains TMB3400, TMB3061 and TMB3130 were characterized in terms of initial D-[U-^14^C]xylose uptake rates in D-glucose- and D-xylose-grown cells. The three strains presented saturation kinetics with the presence of multiple transport systems for D-xylose, as denoted by the non-linear Eadie-Hofstee plots (Figure 4). When grown in D-glucose, the overall *K*_m _for D-xylose uptake was very high (estimated above 300 mM) and similar in the three strains (data not shown). Although *K*_m _values were higher than the maximal sugar concentration tested, the transport capacity for D-xylose at this concentration was estimated for comparative purposes: 33 ± 6 mmol.h^-1^.(g cells)^-1^, 24 ± 8 mmol.h^-1^.(g cells)^-1 ^and 30 ± 5 mmol.h^-1^.(g cells)^-1 ^for TMB3400 (X), TMB3061 (X+A) and TMB3130 (X+A evolved), respectively.

When grown in D-xylose, TMB3400 showed similar kinetic parameters for xylose uptake as when grown in D-glucose (Figure 4). In contrast, TMB3061 and TMB3130 showed higher D-xylose uptake rates (1.5- to twofold) for each tested concentration, which apparently resulted from a lower overall *K*_m _for D-xylose (estimated around 250 mM both for TMB 3061 and TMB 3130 (Figure 4)).

When comparing strains, the transport capacity for xylose in the strains pre-grown in D-glucose (g) or D-xylose (x) was ranked as follows: TMB3061 (g) < TMB3130 (g) < TMB3400 (g) ~ TMB3400 (x) < TMB3061 (x) < TMB3130 (x). Xylose transport was around 1.2- to 1.5-fold higher in TMB 3130 as compared to its parental TMB 3061.

The kinetic parameters for L-[1-^14^C]arabinose uptake were more difficult to evaluate because the uptake capacity was much lower for L-arabinose than for D-xylose. Therefore, only 5 mM and 100 mM concentrations were used in L-arabinose transport experiments with strains TMB3400, TMB3061, TMB3063 and TMB 3130.

**Figure 5:**
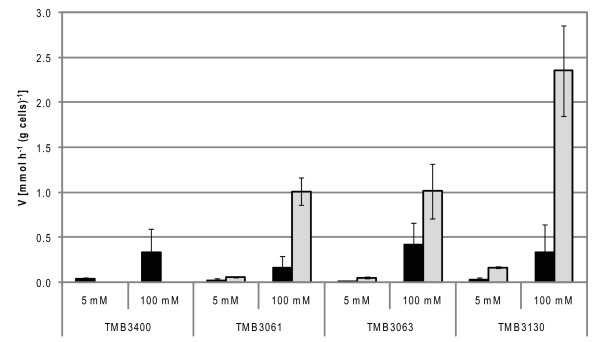
**L-arabinose initial uptake rates in *Saccharomyces cerevisiae *strains engineered for xylose and arabinose utilization**. L-[1-^14^C]Arabinose initial uptake rates of *S. cerevisiae *cells (TMB3400, TMB3061, TMB3063 and TMB3130) grown in D-glucose (black bars) or L-arabinose (grey bars).

When grown in D-glucose all strains showed L-arabinose uptake rates below 0.5 mmol.h^-1^.(g cells)^-1 ^using 100 mM L-arabinose (Figure 5) which was around 10-times lower than xylose uptake under the same conditions. The tendency for L-arabinose uptake was, however, the same as for xylose uptake on glucose-grown cells (g): TMB3061 (g) < TMB3130 (g) < TMB3400 (g) (Figure 5).

When grown in L-arabinose, TMB3061 and TMB3063 showed similar uptake capacities both for 5 mM and 100 mM L-arabinose (0.05 ± 0.01 and 1.0 ± 0.3 mmol.h^-1^.g cells^-1^, respectively; Figure 5). In contrast TMB3130 showed significant improvement in L-arabinose transport when grown in L-arabinose (0.17 ± 0.01 and 2.4 ± 0.5 mmol.h^-1^.g cells^-1^, respectively for 5 mM and 100 mM L-arabinose) (Figure 5), representing two- to threefold higher L-arabinose uptake compared to the parental strain TMB3061.

## Discussion

In the present work, evolutionary engineering was used to improve xylose and arabinose co-utilization in a recombinant industrial *S. cerevisiae *strain carrying the genes from fungal xylose and bacterial arabinose pathways chromosomally integrated in the genome. Both the aerobic growth rate and the final biomass concentration were significantly improved in the evolved strain.

Under anaerobic conditions, the evolved strain TMB3130 displayed higher total anaerobic pentose co-consumption rate as well as the highest ethanol yield. This resulted from an improved conversion of xylose to ethanol (with less xylitol and glycerol as by-products) whereas arabinose was almost stochiometrically converted to arabitol via XR enzyme (Figure 1). We suspect that the use of XR for arabinose conversion to arabitol triggered a shortage of reduced nicotinamide adenine dinucleotide phosphate (NADPH) for the XR reaction, as observed from the increased oxidized nicotinamide adenine dinucleotide phosphate (NADP^+^)-dependent acetate production. Lower NADPH may also have forced XR to use NADH for the conversion of xylose to xylitol, which has been shown to lower xylitol production as a result of a more balanced co-factor utilization between XR and XDH enzymes [17]. In addition, the reduced available NADH would result in lower glycerol production. Overall ethanol production should increase since less carbon is being diverted to glycerol and xylitol by-products.

Molecular and physiological analyses of the evolved isolate TMB3130 revealed that improved transport capacity and initial sugar metabolism contributed to the increased co-utilization of xylose and arabinose. In contrast the molecular basis for the improvements of the haploid laboratory strain have so far not been reported [16]. Comparing isolate TMB3130 and its parental strains at the gene level did not reveal any mutation in the heterologous genes of the pentose converting enzymes. However, the parental strain contained several mutations in *AraA *and few in *AraB*, as compared to the original bacterial sequence. This may indicate a high selective pressure on the first enzymes of the arabinose utilization pathway during arabinose consumption and would confirm previous indications on the pivotal role of L-arabinose isomerase in arabinose-engineered *S. cerevisiae *[7]. Also two silent mutations in *B. subtilis AraA *were shifted towards codon usage found at increased frequencies in highly expressed genes [18].

In the evolved strain TMB3130, the XR and XDH activities increased 5.5- and 3.5-fold, respectively, as compared to the parental strain TMB3061. This concomitant increase in activity associated with unchanged gene sequences suggest that the higher XR and XDH activities result from one or several duplications on the genome of the integrative vector that carry *XYL1 *and *XYL2 *genes. XR and XDH levels were also higher than for the original strain TMB3400, although xylose consumption rates were in the same range. Therefore, the loss of xylose consumption capacity in strain TMB3061 was not associated with decreased XR-XDH activity whereas the recovery of xylose consumption capacity in the evolved strain was. It suggests that *S. cerevisiae *is able to use different strategies to recover a phenotype under selection pressure.

Under glucose limitation or absence of glucose, high-affinity glucose transporters are induced [19]. This correlates with the higher pentose uptake rate that was observed when strains were pre-grown on xylose or arabinose instead of glucose since hexose transporters have been shown to also transport pentoses: Hxt4p, Hxt5p, Hxt7p and Gal2p can transport xylose [20,21] whereas Gal2p can also transport arabinose [22]. Xylose and arabinose transport was improved in the evolved strain TMB3130, 1.2- to 1.5-fold and two- to threefold, respectively. This suggests an improvement in pentose uptake rate through evolution and confirms that continuous cultivation under substrate limitation tends to generate an evolved population with improvement at the level of nutrient transport [12,13]. Specific xylose and arabinose transporters have been reported in yeast [23] and overexpression of one or several of these transporters may further improve simultaneous pentose fermentation.

Despite significant improvement, the xylose and arabinose co-utilization rate of the evolved industrial strain TMB3130 was three- and fivefold lower, respectively, than what was achieved with the evolved laboratory strain [16]. TMB3130 was derived from TMB3400 that is at least diploid and originates from a cross between wine-making strains [24]. It has been suggested that it is more difficult to obtain substantial strain improvement by evolution of industrial *S. cerevisiae *strains compared to haploid laboratory strains, because of the intrinsic genetic robustness that result from the polyploidy of industrial strains [11,14]. This would explain the limited success of evolutionary approaches with industrial *S. cerevisiae *strains for bioethanol production [14,25].

The evolutionary engineering protocol used to increase the consumption rate of xylose and arabinose in the laboratory *S. cerevisiae *strain [16] and in the currently reported industrial *S. cerevisiae *strain differed significantly in the selection method. The laboratory strain was selected through repeated batch cultivations in media alternating combinations of glucose, xylose and arabinose sugars, after limited success in evolving the parent under chemostat conditions [16]. In contrast, the industrial strain was improved by increasing the dilution rate in a continuous cultivation with a defined medium containing a mixture of xylose and arabinose as sole carbon sources. The industrial strain carried all genes from the heterologous pentose pathways on the genome whereas the engineered CEN.PK strain combined integrated and plasmid-born genes. On the one hand multicopy plasmids may favour fine tuning of the gene copy number to match the required expression levels, on the other hand it may be more difficult to reach a stable phenotype for mixed sugar utilization unless careful selection pressure (such as successive cultivation on alternative sugars) is applied. It has previously been reported that a strain harbouring a multicopy plasmid was more prone to evolution for improved aerobic growth on xylose than the corresponding strain with all genes integrated on the genome [13]. Therefore, one can speculate that plasmids may increase the strain instability, thereby increasing the mutation or recombination rate and favour evolution.

## Conclusions

An industrial yeast strain was evolved for improved xylose and arabinose co-utilization through continuous cultivation at increasing dilution rate with xylose and arabinose as sole carbon sources. The xylose and arabinose consumption rates increased both under aerobic and anaerobic conditions. As a result, the aerobic growth rate and the final cell mass on xylose and arabinose increased. Under anaerobic conditions, the ethanol yield increased at the expense of the xylitol and glycerol yields, whereas arabinose was almost entirely converted to arabitol. Increased transport of xylose and arabinose as well as increased levels of xylose converting enzymes most likely contributed to the improvement of pentose utilization. To the best of our knowledge, this is the first report on improved mixed-pentose utilization that characterizes the molecular mechanisms for the evolutionary engineered strain.

## Methods

### Strains

Yeast strains used in the study are summarized in Table 1. All strains were stored in 15% glycerol at -80°C.

### Aerobic cultivations

Aerobic cultivation was performed on YNB (6.7 g L^-1 ^Difco YNB without amino acids; Becton, Dickinson and Company, MD, USA). YNB medium was supplemented with 50 g L^-1 ^arabinose, 50 g L^-1 ^xylose or 20 g L^-1 ^glucose unless otherwise stated. For aerobic growth determinations, pre-cultivation conditions were normalized by growing the cells in YNB medium with 20 g L^-1 ^glucose until late exponential phase from which cell culture was used to inoculate YNB medium with arabinose or xylose at an OD _620 nm _of 0.2. Double concentration of YNB was used when the sugar concentration was above 20 g L^-1^. YNB medium was buffered with potassium hydrogen phthalate (10.21 g L^-1 ^phthalate, 2.1 g L^-1 ^KOH) at pH 5.5 [26]. The cultures were grown in cotton-stoppered baffled shake-flasks with starting medium volume of 10% of the flask volume. Temperature was controlled at 30°C and shaking was set at 180-200 rpm (Gallenkamp INR-200, Leicester, UK). Cell growth was monitored by determining the optical density (OD _620 nm_) with a Hitachi U-1800 Spectrophotometer (Hitachi Ltd, Tokyo, Japan). YPD (10 g L^-1 ^yeast extract, 20 g L^-1 ^peptone, 20 g L^-1 ^glucose, 20 g L^-1 ^agar) or YNB (6.7 g L^-1^, 30 g L^-1 ^agar) plates were used for cell culture from frozen stocks, for inoculation and for recovery of cells from the chemostat or isolation of the evolvants.

### Continuous culture for evolutionary engineering

The pre-culture of TMB3061 cells was grown aerobically using YNB medium with 20 g L^-1 ^glucose. The cells were harvested and mixed with the fermentation medium to an OD _620 nm _of 0.5. The continuous cultivation was performed in a 250 mL in-house-built jacketed bioreactor on defined medium [27] that contained 0.05% (vol/vol) antifoam (Dow Corning^® ^Antifoam RD Emulsion; BDH Laboratory Supplies, Dorset, UK). For the initial batch culture, 150 mL of working volume was used with 500 rpm magnetic stirring and for the chemostat culture the volume was reduced to approximately 100 mL with 250 rpm stirring. Air was supplied by sparging 0.8 L air gas min^-1 ^controlled with a gas flow-meter (Bronkhorst, HI-TECH, Ruurlo, The Netherlands). The temperature was set at 30°C and pH was maintained at 5.5 by automatic addition of 0.3 M KOH. Outlet gases from the culture were followed with a Carbon Dioxide and Oxygen Monitor type 1308 (Brüel and Kjaer, Copenhagen, Denmark).

In the initial batch culture, 10 g L^-1 ^glucose and 10 g L^-1 ^xylose were used as carbon sources. Then the carbon source in the feed was 10 g L^-1 ^xylose, followed by a mixture of 5 g L^-1 ^xylose and 5 g L^-1 ^arabinose. The initial dilution rate (0.02 h^-1^) was increased stepwise every time the culture reached a steady state. The absence of contamination was checked by microscopic examination.

### Anaerobic co-fermentation of glucose and pentose

For the characterization of evolved strains, pre-cultures were grown in defined mineral medium [27] with 20 g L^-1 ^glucose buffered to pH 5.5 with potassium hydrogen phthalate [26]. A first pre-culture grown overnight in a test tube was used to inoculate a second 100 mL pre-culture in a baffled 1 L flask. Cells from late exponential phase were harvested, washed twice with 0.9% NaCl and inoculated at an OD _620 nm _of 0.2. Fermentation was conducted in 3 L Applikon^® ^Bio Reactors (Applikon, Schiedam, The Netherlands) with a working volume of 1.5 L. Defined mineral medium [27] supplemented with a mixture of 20 g L^-1 ^glucose, 20 g L^-1 ^xylose and 20 g L^-1 ^arabinose was used. For anaerobic fermentation 0.4 g L^-1 ^Tween-80 and 0.01 g L^-1 ^ergosterol were added to the medium. Temperature was set at 30°C, agitation at 200 rpm and pH was maintained at 5.5 by automatic addition of 3 M KOH. Anaerobic conditions were obtained by sparging nitrogen (AGA Gas, Sundbyberg, Sweden) at a flow rate of 0.2 L min^-1^. The experiments were performed at least in duplicate.

### Analysis of metabolites and cell dry weight

The concentrations of xylose, glucose, acetic acid, glycerol, xylitol and ethanol were determined in the filtered sample supernatant by high performance liquid chromatography (Beckman Instruments, CA, USA) using Aminex HPX-87H ion exchange columns (Bio-Rad, CA, USA) at 45°C with 5 mM H_2_SO_4 _as mobile phase and at a flow rate of 0.6 mL min^-1^. A series of three connected columns was used for separation of glucose, xylose and arabinose and their corresponding fermentation by-products when present in the same sample. Arabinose, arabitol and xylitol were separated using a HPX-87P ion exchange column (Bio-Rad, CA, USA) at 85°C with a mobile phase of water at a flow rate of 0.5 mL min^-1^. A RID-6A refractive index detector (Shimadzu, Kyoto, Japan) was used for the detection of all the compounds. Ethanol concentration was calculated from the degree of reduction balance.

Cell dry weight was determined in triplicates by filtering 5 mL culture through a pre-weighed hydrophilic polyethersulfone filter (PALL, Life Sciences, Michigan, USA).

### Enzyme activities

XR and XDH assays were performed with an Ultrospec 2100 pro spectrophotometer (Amersham Biosciences, Uppsala, Sweden). Freshly streaked cells grown in YNB plates supplemented with 20 g L^-1 ^glucose were inoculated in tubes containing YNB liquid medium with 20 g L^-1 ^glucose. Overnight grown cells were then used to inoculate shake flask cultures with 20 g L^-1 ^glucose. Cells were harvested in exponential phase and washed twice with water. Y-PER reagent (Pierce Biotechnology, IL, USA) was used to extract proteins. The protein concentration was determined with the Coomassie Plus protein assay reagent (Pierce, IL, USA) with bovine serum albumine as standard. XR activity was measured as previously described [28,29]. For the XDH assay [30] triethanolamine buffer at pH 7 [31] was used. Experiments were performed in biological triplicates and with duplicates of different concentrations of the extracted proteins. Arabinose isomerase activity could not be determined because of the unavailability of commercial reagents.

### Genetic analysis

In order to verify that the isolates originated from TMB3061, the overexpressed endogenous xylulokinase gene was amplified by PCR using primers binding to the heterologous *PGK1 *promoter and to the coding sequence of *XKS1 *gene. Yeast genomic DNA was extracted with Easy-DNA Kit (Invitrogen, Groningen, The Netherlands) or by phenol/chloroform method. The *AraA, AraB*, *AraD, XYL1 *and *XYL2 *genes were sequenced in strains TMB3061 and TMB3130. The *Pfu *DNA polymerase or high fidelity PCR enzyme mix that were used for PCR amplification were obtained from Fermentas (Vilnius, Lithuania). The QIAquick^® ^Gel Extraction Kit (Qiagen GmbH, Hilden, Germany) was used for purification of DNA fragments from agarose gel. DNA sequencing and oligonucleotide synthesis was performed at Eurofins MWG Operon (Martinsried, Germany).

### Transport kinetics

Cells were inoculated in shake flasks with medium (volume equal to one-fifth of flask capacity) containing YNB (without amino acids) and D-glucose, D-xylose or L-arabinose (20 g L^-1^). Cells were grown aerobically at 30°C, 180 rpm, to exponential phase (OD _640 nm _= 1). Initial uptake rates were determined at 25°C [32], using D-[U-^14^C]glucose, D-[U-^14^C]xylose (GE Healthcare, Buckinghamshire, UK) or L-[1-^14^C]arabinose (Moravek Biochemicals Inc, CA, USA). D-[U-^14^C]Xylose uptake measurements were performed using a sugar concentration range between 0.5 mM and 100 mM (final concentrations) and a period of 10 s. L-[1-^14^C]arabinose uptake measurements were performed using sugar concentrations of 5 mM and 100 mM (final concentrations) and a period of 5-10 s.

## Abbreviations

OD: optical density; PCR: polymerase chain reaction; XDH: xylitol dehydrogenase; XR: xylose reductase; YNB: yeast nitrogen base; YPD: yeast peptone glucose.

## Competing interests

The authors declare that they have no competing interests.

## Authors' contributions

CF and VSN contributed equally. RGS participated in the experimental design, performed the physiological analysis of the mutant strains, participated in screening, fermentation experiments, initial experiments of transport assays, sequencing, enzymatic assays, data analysis and drafted the manuscript. KK participated in initial planning of the study and in the experimental design, performed chemostat cultivations and helped to draft the manuscript. CF designed and performed transport assays and commented the manuscript. VSN participated in the screening and physiological analysis of mutant strains. JRMA, OB and CUL participated in initial planning of the study and performed chemostat cultivations. MB participated in fermentations performance. BHH participated in initial planning of the study and in the experimental design and edited the manuscript. MFGG planned the study, participated in the experimental design and edited the manuscript.

## References

[B1] Hayn MSW Klinger R Steinmüller H Sinner M Esterbauer H Saddler JN Basic research and pilot studies on the enzymatic conversion of lignocellulosics Bioconversion of Forest and Agricultural Plant Residues 1993 Wallingford: CAB International 33 72

[B2] Hahn-Hägerdal B Karhumaa K Jeppsson M Gorwa-Grauslund MF Metabolic engineering for pentose utilization in *Saccharomyces cerevisiae* Biofuels 2007 108 Berlin/Heidelberg: Springer 147 177 full_text 10.1007/10_2007_062 17846723

[B3] Matsushika A Inoue H Kodaki T Sawayama S Ethanol production from xylose in engineered *Saccharomyces cerevisiae *strains: current state and perspectives Applied Microbiology and Biotechnology 2009 84 1 37 53 10.1007/s00253-009-2101-x 19572128

[B4] Karhumaa K Garcia Sanchez R Hahn-Hägerdal B Gorwa-Grauslund MF Comparison of the xylose reductase-xylitol dehydrogenase and the xylose isomerase pathways for xylose fermentation by recombinant *Saccharomyces cerevisiae* Microbial Cell Factories 2007 6 5 10.1186/1475-2859-6-5 17280608 PMC1797182

[B5] Becker J Boles E A modified *Saccharomyces cerevisiae *strain that consumes L-Arabinose and produces ethanol Appl Environ Microb 2003 69 7 4144 4150 10.1128/AEM.69.7.4144-4150.2003 PMC165137 12839792

[B6] Wisselink HW Toirkens MJ del Rosario Franco Berriel M Winkler AA van Dijken JP Pronk JT van Maris AJA Engineering of *Saccharomyces cerevisiae *for efficient anaerobic alcoholic fermentation of L-Arabinose Appl Environ Microb 2007 73 15 4881 4891 10.1128/AEM.00177-07 17545317 PMC1951023

[B7] Wiedemann B Boles E Codon-optimized bacterial genes improve L-Arabinose fermentation in recombinant *Saccharomyces cerevisiae* Appl Environ Microb 2008 74 7 2043 2050 10.1128/AEM.02395-07 18263741 PMC2292587

[B8] Richard P Verho R Putkonen M Londesborough J Penttilä M Production of ethanol from L-arabinose by *Saccharomyces cerevisiae *containing a fungal L-arabinose pathway FEMS Yeast Research 2003 3 2 185 189 10.1016/S1567-1356(02)00184-8 12702451

[B9] Bettiga M Bengtsson O Hahn-Hägerdal B Gorwa-Grauslund MF Arabinose and xylose fermentation by recombinant *Saccharomyces cerevisiae *expressing a fungal pentose utilization pathway Microbial Cell Factories 2009 8 40 10.1186/1475-2859-8-40 19630951 PMC2720912

[B10] Karhumaa K Wiedemann B Hahn-Hägerdal B Boles E Gorwa-Grauslund MF Co-utilization of L-arabinose and D-xylose by laboratory and industrial *Saccharomyces cerevisiae *strains Microbial Cell Factories 2006 5 18 10.1186/1475-2859-5-18 16606456 PMC1459190

[B11] Sauer U Evolutionary engineering for industrially important microbial phenotypes Advances in Biochemical Engineering/Biotechnology 2001 73 129 169 full_text 11816810 10.1007/3-540-45300-8_7

[B12] Kuyper M Toirkens MJ Diderich JA Winkler AA van Dijken JP Pronk JT Evolutionary engineering of mixed-sugar utilization by a xylose-fermenting *Saccharomyces cerevisiae *strain FEMS Yeast Research 2005 5 10 925 934 10.1016/j.femsyr.2005.04.004 15949975

[B13] Pitkänen JP Rintala E Aristidou A Ruohonen L Penttilä M Xylose chemostat isolates of *Saccharomyces cerevisiae *show altered metabolite and enzyme levels compared with xylose, glucose, and ethanol metabolism of the original strain Applied Microbiology and Biotechnology 2005 67 6 827 837 10.1007/s00253-004-1798-9 15630585

[B14] Sonderegger M Jeppsson M Larsson C Gorwa-Grauslund MF Boles E Olsson L Spencer-Martins I Hahn-Hägerdal B Sauer U: Fermentation performance of engineered and evolved xylose-fermenting *Saccharomyces cerevisiae *strains Biotechnology and Bioengineering 2004 87 1 90 98 10.1002/bit.20094 15211492

[B15] Sonderegger M Sauer U Evolutionary engineering of *Saccharomyces cerevisiae *for anaerobic growth on xylose Appl Environ Microb 2003 69 4 1990 1998 10.1128/AEM.69.4.1990-1998.2003 PMC154834 12676674

[B16] Wisselink HW Toirkens MJ Wu Q Pronk JT Van Maris AJA Novel evolutionary engineering approach for accelerated utilization of glucose, xylose and arabinose mixtures by engineered *Saccharomyces cerevisiae* Appl Environ Microb 2009 75 4 907 914 10.1128/AEM.02268-08 PMC2643596 19074603

[B17] Jeppsson M Johansson B Hahn-Hägerdal B Gorwa-Grauslund MF Reduced oxidative pentose phosphate pathway flux in recombinant xylose-utilizing *Saccharomyces cerevisiae *strains improves the ethanol yield from xylose Appl Environ Microb 2002 68 4 1604 1609 10.1128/AEM.68.4.1604-1609.2002 PMC123863 11916674

[B18] Sharp PM Cowe E Synonymous codon usage in *Saccharomyces cerevisiae* Yeast 1991 7 657 678 10.1002/yea.320070702 1776357

[B19] Kruckeberg AL The hexose transporter family of *Saccharomyces cerevisiae* Arch Microbiol 1996 166 5 283 292 10.1007/s002030050385 8929273

[B20] Hamacher T Becker J Gárdonyi M Hahn-Hägerdal B Boles E Characterization of the xylose-transporting properties of yeast hexose transporters and their influence on xylose utilization Microbiology 2002 148 Pt 9 2783 2788 12213924 10.1099/00221287-148-9-2783

[B21] Sedlak M Ho NWY Characterization of the effectiveness of hexose transporters for transporting xylose during glucose and xylose co-fermentation by a recombinant *Saccharomyces *yeast Yeast 2004 21 8 671 684 10.1002/yea.1060 15197732

[B22] Cirillo VP Galactose transport in *Saccharomyces cerevisiae*. I. nonmetabolized sugars as substrates and inducers of galactose transport system J Bacteriol 1968 95 5 1727 1731 5650080 10.1128/jb.95.5.1727-1731.1968 PMC252203

[B23] Leandro MJ Fonseca C Gonçalves P Hexose and pentose transport in ascomycetous yeasts: an overview FEMS Yeast Research 2009 9 4 511 525 fingerprinting and karyotyping i 10.1111/j.1567-1364.2009.00509.x 19459982

[B24] Vander Westhuizen TJ Pretorius IS The value of electrophoretic fingerprinting and karyotyping in wine yeast breeding programs Antonie Van Leeuwenhoek Int J Gen Molec Microbiol 1992 61 4 249 257 10.1007/BF00713932 1497329

[B25] Heer D Sauer U Identification of furfural as a key toxin in lignocellulosic hydrolysates and evolution of a tolerant yeast strain Microbial Biotechnol 2008 1 6 497 506 10.1111/j.1751-7915.2008.00050.x PMC3815291 21261870

[B26] Hahn-Hägerdal B Karhumaa K Larsson CU Gorwa-Grauslund M Görgens J van Zyl WH Role of cultivation media in the development of yeast strains for large scale industrial use Microbial Cell Factories 2005 4 31 10.1186/1475-2859-4-31 16283927 PMC1316877

[B27] Jeppsson M Bengtsson O Franke K Lee H Hahn-Hägerdal B Gorwa-Grauslund MF The expression of a *Pichia stipitis *xylose reductase mutant with higher K(M) for NADPH increases ethanol production from xylose in recombinant *Saccharomyces cerevisiae* Biotechnology and Bioengineering 2006 93 4 665 673 10.1002/bit.20737 16372361

[B28] Eliasson A Christensson C Wahlbom CF Hahn-Hägerdal B Anaerobic xylose fermentation by recombinant *Saccharomyces cerevisiae *carrying *XYL1*, *XYL2*, and *XKS1 *in mineral medium chemostat cultures Appl Environ Microb 2000 66 8 3381 3386 10.1128/AEM.66.8.3381-3386.2000 10919795 PMC92159

[B29] Smiley KL Bolen PL Demonstration of D-xylose reductase and D-xylitol dehydrogenase in *Pachysolen tannophilus* Biotech Lett 1982 4 607 610 10.1007/BF00127793

[B30] Rizzi MHK Erlemann P Bui-Thahn NA Dellweg H Purification and properties of the NAD^+^-xylitol-dehydrogenase from the yeast *Pichia stipitis* J Fermentation Bioengineering 1989 67 20 24 10.1016/0922-338X(89)90080-9

[B31] Wahlbom CF van Zyl WH Jönsson LJ Hahn-Hägerdal B Otero RR Generation of the improved recombinant xylose-utilizing *Saccharomyces cerevisiae *TMB 3400 by random mutagenesis and physiological comparison with *Pichia stipitis *CBS 6054 FEMS Yeast Res 2003 3 3 319 326 10.1016/S1567-1356(02)00206-4 12689639

[B32] Fonseca C Romão R Rodrigues de Sousa H Hahn-Hägerdal B Spencer-Martins I L-Arabinose transport and catabolism in yeast FEBS J 2007 274 14 3589 3600 10.1111/j.1742-4658.2007.05892.x 17627668

